# Neonatal Calf Infection with Respiratory Syncytial Virus: Drawing Parallels to the Disease in Human Infants

**DOI:** 10.3390/v4123731

**Published:** 2012-12-13

**Authors:** Randy E. Sacco, Jodi L. McGill, Mitchell V. Palmer, John D. Lippolis, Timothy A. Reinhardt, Brian J. Nonnecke

**Affiliations:** 1 Ruminant Diseases and Immunology Unit, National Animal Disease Center, Agricultural Research Service, United States Department of Agriculture, Ames, Iowa, 50010, USA; E-Mails: Jodi.McGill@ars.usda.gov (J.L.M.); John.Lippolis@ars.usda.gov (J.D.L.); Tim.Reinhardt@ars.usda.gov (T.A.R.); Brian.Nonnecke@ars.usda.gov (B.J.N.); 2 Infectious Bacterial Diseases Research Unit, National Animal Disease Center, Agricultural Research Service, United States Department of Agriculture, Ames, Iowa, 50010, USA; E-Mail: Mitchell.Palmer@ars.usda.gov

**Keywords:** Bovine respiratory syncytial virus, human respiratory syncytial virus, innate immunity, adaptive immunity, vaccine

## Abstract

Respiratory syncytial virus (RSV) is the most common viral cause of childhood acute lower respiratory tract infections. It is estimated that RSV infections result in more than 100,000 deaths annually worldwide. Bovine RSV is a cause of enzootic pneumonia in young dairy calves and summer pneumonia in nursing beef calves. Furthermore, bovine RSV plays a significant role in bovine respiratory disease complex, the most prevalent cause of morbidity and mortality among feedlot cattle. Infection of calves with bovine RSV shares features in common with RSV infection in children, such as an age-dependent susceptibility. In addition, comparable microscopic lesions consisting of bronchiolar neutrophilic infiltrates, epithelial cell necrosis, and syncytial cell formation are observed. Further, our studies have shown an upregulation of pro-inflammatory mediators in RSV-infected calves, including IL-12p40 and CXCL8 (IL-8). This finding is consistent with increased levels of IL-8 observed in children with RSV bronchiolitis. Since rodents lack IL-8, neonatal calves can be useful for studies of IL-8 regulation in response to RSV infection. We have recently found that vitamin D in milk replacer diets can be manipulated to produce calves differing in circulating 25-hydroxyvitamin D_3_. The results to date indicate that although the vitamin D intracrine pathway is activated during RSV infection, pro-inflammatory mediators frequently inhibited by the vitamin D intacrine pathway *in vitro* are, in fact, upregulated or unaffected in lungs of infected calves. This review will summarize available data that provide parallels between bovine RSV infection in neonatal calves and human RSV in infants.

## 1. Importance of Human and Bovine Respiratory Syncytial Viruses

Human (hRSV) and bovine (bRSV) respiratory syncytial viruses are closely related viruses that are among the leading causes of acute serious lower respiratory infection (ALRI) in young children and calves, respectively. While estimated mortality rates in developing countries are low (<0.02%), globally, it is estimated that new episodes of hRSV-associated disease in children younger than 5 years of age exceed 33 million annually, with more than 100,000 resultant deaths [1]. Severe hRSV infections during the first three years of life are frequently followed by recurrent episodes of childhood wheezing or asthma [2,3]. In addition, hRSV is increasingly seen as an important cause of morbidity and mortality in elderly adults [4,5] and in immunocompromised patients [6].

BRSV is a cause of enzootic pneumonia in young dairy calves and summer pneumonia in nursing beef calves. In fact, worldwide estimates suggest the frequency of bRSV infections in some dairy and beef herds exceeds 50% [7]. Furthermore, in combination with other viral and bacterial pathogens, bRSV plays a significant role in shipping fever or bovine respiratory disease complex (BRDC), the most prevalent cause of morbidity and mortality among feedlot cattle. Even in cases where animals do not succumb to the disease, there can be long-term losses in performance. This includes reductions in feed efficiency and rate of gain in the feedlot, as well as reproductive performance, milk production, and longevity in the breeding herd. As a result, economic costs to the cattle industry from BRDC have been estimated to approach $1 billion annually due to death losses, reduced performance, and costs of vaccinations and treatment modalities.

## 2. Historical Perspectives on Respiratory Syncytial Viruses

In 1956, RSV was originally isolated as a causal agent of chimpanzee coryza [8]. However, at the time this virus was further linked with respiratory illness in a human working with the coryza agent that had close contact with the chimpanzees. The next year, there was a reported isolation of a virus from infants with respiratory illness that was indistinguishable from the chimpanzee coryza agent [9,10]. The first study to propose an involvement of RSV in respiratory disease in cattle came more than a decade later. Doggett *et al.* [11] found bovine sera that contained neutralizing antibody against hRSV, suggesting a similar virus might exist in cattle. In the early 1970s, two reports from respiratory disease outbreaks in Switzerland [12] and England [13] identified viruses isolated from cattle that were closely related to hRSV. At the same time, Inaba *et al.* [14] reported the isolation of what appeared to be a new virus from cases of acute BRDC that was initially referred to as Nomi virus, but which they subsequently identified as bRSV [15].

## 3. Age and Seasonal Affects

The most severe ALRI cases due to hRSV occur in infants and children less than 1 year of age, especially those born premature or with underlying cardiopulmonary conditions [4,5]. In the USA, by 24 months of age, nearly all children have been infected at least once with hRSV, and approximately half have experienced two infections [16]. BRSV-associated disease is most pronounced in calves less than 6 months of age, and infection can occur even in the presence of maternal antibodies. It has been estimated that more than 70% of calves have seroconverted by the age of 12 months [17]. As with humans, re-infections in calves are common. Seasonal periodicity is seen with hRSV and bRSV, with most common occurrences of infections in the fall and winter months [18].

## 4. RSV Viral Proteins

HRSV and bRSV belong to the family *Paramixoviridae*, subfamily Pneumoviridae, and genus Pneumovirus. Pneumoviruses are single-stranded, negative-sense RNA viruses with a genome of approximately 15.2 kb. The RSV viral RNA is transcribed into 10 major subgenomic mRNAs encoding 11 proteins, due to the M2 gene encoding two proteins. Associated with the genomic RNA, are nucleocapsid (N), phosphoprotein (P), large polymerase (L), and associated proteins, transcriptional anti-termination factor M2-1 and RNA regulatory protein M2-2. There are 3 transmembrane surface glycoproteins, attachment (G), fusion (F), and small hydrophobic (SH). A non-glycosylated matrix or membrane protein, M, is associated with the inner face of the envelope. Finally, there are 2 non-structural proteins that accumulate in infected cells NS1, NS2.

HRSV has been classified into two subgroups, A and B, based on antigenic and genetic differences [19,20]. BRSV isolates can be classified into subgroups based on reactivity of mAb to the G protein [21], although these may represent variants of a single major antigenic group [21,22]. The F protein is a type I viral fusion protein synthesized as a precursor that is proteolytically cleaved by furin into disulfide-linked fragments [23]. Among the hRSV subgroups, the cellular attachment G protein, a type II integral membrane protein, is more divergent than the F protein [24]. Although bRSV isolates possess antigenically heterogeneous G proteins, the nucleotide sequences are less variable than for hRSV [21,25]. The F and G glycoproteins contain the predominant neutralization and protective epitopes. For both hRSV and bRSV, the attachment protein is a major target of the host anti-RSV antibody response [26] and specific regions of the protein may be under immune selection [27]. However, immunological pressures may differ between bRSV and hRSV as suggested by differences in the variability of the central hydrophobic region of the G protein of these viruses [28].

## 5. Experimental Bovine RSV in Calves and Similarities to Lesions seen in Human Infants

We have used bRSV strain 375 for inoculation of neonates in our studies [29,30,31]. The inoculum was prepared from virus stock re-isolated from the lung of an infected animal and passaged less than 4 times on bovine turbinate cells. Our bRSV aerosol challenge model [31] was adapted from that described by Woolums *et al.* [32]. Briefly, the challenge inoculum is delivered by nebulization into a mask covering the nostrils and mouth. The nebulization apparatus consists of a compressed air tank, a jet nebulizer, and a mask (Trudell Medical International, London, Ontario, Canada) modified to fit calves. Compressed air (25 lb/in^2^) is used to jet nebulize the challenge inoculum directly into a holding reservoir. Upon installation, the nebulized inoculum is inhaled through a one-way valve into the mask and directly into the nostrils. Each calf received a 5 ml challenge inoculum containing approximately 10^4^ TCID_50_/ml of bRSV strain 375 during the nebulization period of 10–15 min.

**Figure 1:**
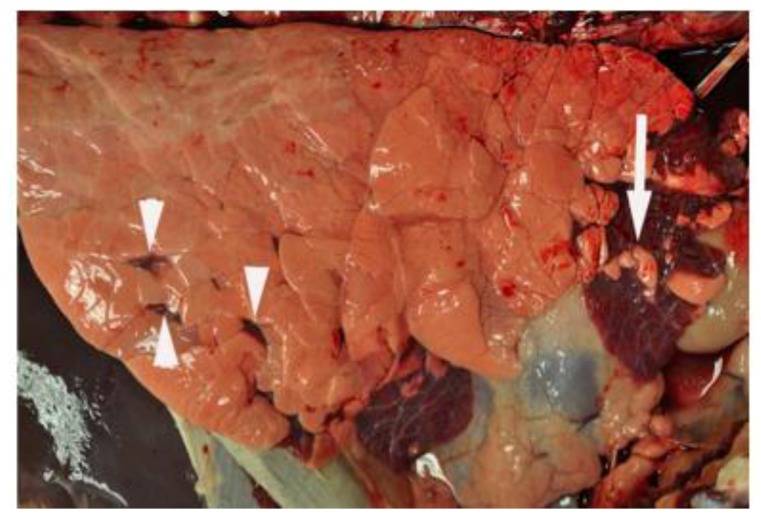
**Lung from an RSV-infected calf at day 7 post-infection.** Note multifocal to coalescing areas of plum-red consolidation in cranial and ventral aspects of right cranial and middle lobes. Consolidated areas surround and divide foci of pale pink hyperinflated lung (arrow). Scattered multifocal areas of consolidation are also present in the ventral third of the right caudal lobe (arrowheads). Original figure: [31].

**Figure 2:**
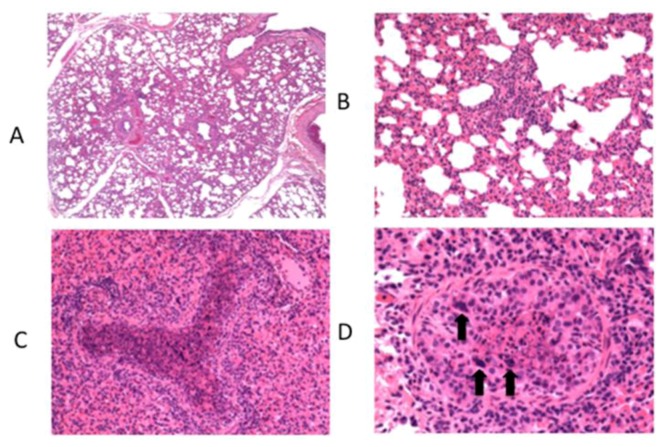
Histological lesions observed in lung of calves after experimental infection with bovine RSV. Calves were challenged via aerosol with bovine RSV. On day 7 post-infection, samples of lung were collected for histological evaluation. A representative image from a single calf is shown. (**A**) Moderate interstitial thickening and wide interlobular septae due to edema. Original magnification, 4X. (**B**) Alveolar septae are thickened due to cellular infiltrates found to be macrophages, lymphocytes and lesser numbers of neutrophils when viewed at higher magnification. Original magnification, 20X. (**C**) Bronchioles are filled with neutrophils, sloughed epithelial cells, and necrotic cell debris. Original magnification, 20X. (**D**) There is partial to complete loss of bronchiolar epithelial cells with attenuation of remaining cells. In some bronchioles, epithelial cells form multinucleated syncytial cells (arrows). Original magnification, 40X. Figure adapted from: [31].

At necropsy, performed on day 7 post infection, gross lesions typically consist of bilateral, multifocal, firm, plum-red areas of consolidation that are of variable size and depressed compared to the adjacent normal appearing lung (Figure 1). Lesions are most frequently observed in cranioventral lung lobes. On cut surface, areas of consolidation are well delineated from adjacent normal lung. In some cases, areas of consolidation surround and divide regions of pink, hyperinflated lung. Significant microscopic lesions are apparent with representative photomicrographs shown (Figure 2). Microscopically, interlobular septa are expanded by clear space interpreted to be edema (Figure 2A). Alveolar septa are thickened due to infiltrates of macrophages, lymphocytes, and lesser numbers of neutrophils (Figure 2B). Intralesional bronchioles are filled with neutrophils, sloughed epithelial cells, and necrotic cellular debris (Figure 2C). Bronchiolar epithelial cell necrosis results in attenuation of remaining epithelial cells, or complete loss of airway epithelium. In some bronchioles, epithelial cells form multinucleated syncytial cells (Figure 2D). These lesions are similar to those described by others following experimental bRSV challenge.

In the rare instances where histopathological evaluation is available from fatal hRSV cases, it was noted that airway lumina contain cellular debris from leukocytes and sloughed epithelial cells, with fibrin and a minor amount of mucin [33,34,35]. Submucosal edema and peribronchiolar infiltrates, consisting predominately of mononuclear cells with minor numbers of neutrophils, further obstructed the airways. With interstitial pneumonia, there was evidence of significant alveolar involvement including edema and cellular infiltration, as well as epithelial cell attenuation or loss.

## 6. Innate Immunity to RSV

Critical to the induction of the innate immune response are pattern recognition receptors (PRRs) that recognize evolutionarily conserved pathogen-associated molecular patterns (PAMPs). Recognition of viral PAMPs involves at least three distinct classes of PRRs, toll-like receptors (TLRs), retinoic acid inducible gene-I (RIG-I)-like receptors (RLRs), and nucleotide-binding oligomerization domain (NOD)-like receptors (NLRs). Involvement of several TLRs has been described for hRSV infection, including TLR2, -3, -4, -7 and -8 [36,37]. Ligation of PRRs including TLR3, -7 and -8, and RLRs activates interferon regulatory factors (IRFs). These IRFs, in particular IRF3 and 7, interact with other transcription factors to control expression of type I IFNs and related molecules. Inactive IRFs reside in the cytoplasm in a latent form [38]. Viral infection triggers phosphorylation of serine and threonine residues and nuclear translocation of IRF3 and IRF7 where they associate with each other and complex with other co-activators (e.g., CREB-binding protein) to form a transcriptional complex that binds to promoter regions of type I IFN genes to activate transcription [39]. Virus-induced type I IFNs bind to the type I IFN receptor (IFNAR) activating Jak/STAT signaling leading to the upregulation of IFN-stimulated target genes (ISGs). Among ISGs are a number of antiviral mediators, including Mx, PKR, and 2'-5' oligoadenylate synthetase.

*Paramixoviruses* are known to utilize different mechanisms to affect IFN signaling. hRSV and bRSV have evolved strategies to inhibit the IFN-induced cellular response that are dependent upon nonstructural (NS) proteins. As is characteristic of pneumoviruses, these viruses have two genes that encode for NS proteins. It has been shown that NS1 and NS2 proteins cooperatively mediate resistance of bRSV and hRSV to IFN-stimulated responses in a species-specific manner [40,41]. In fact, the precise mechanism whereby type I IFN responses are altered, varies between these viruses. In the case of bRSV, NS proteins block phosphorylation and activation of IRF3 [42]. By comparison, hRSV NS1 and NS2 modulation of type I IFN responsiveness involves inhibition of Stat2 expression [43].

In addition to induction of IRFs, ligation of PRRs by viral PAMPs stimulates the release of inflammatory mediators. It has been shown that RSV induces cytokine/chemokine production in airway epithelial cells via signaling through TLR3 and RLRs, which have been linked to distinct pathways controlling NF-κB activation [44]. In addition to respiratory epithelial cells, RSV infection induces inflammatory cytokines in antigen-presenting cells, the kinetics of which differs between cytokines. For example, we found peak induction of IL-1β and IL-12p40 mRNA in alveolar macrophages occurs on day 3 post-infection in the neonatal bRSV model, whereas IL-6 mRNA was higher at day 5 than day 3 of infection [29]. Recently, we have shown a significant upregulation of the innate chemokine IL-8 in lesioned lungs of bRSV-infected calves on day 7 post infection [31]. Importantly, our results fit well with data showing that IL-8 is elevated in the respiratory tract of children with hRSV bronchiolitis. Rodents lack a *bona fide* homologue of IL-8, although mice have what are considered to be functional homologues, CXCL1 (GRO/KC), CXCL2 (MIP-2) and CXCL5-6 (LIX), which belong to the same major cluster of chemokines. It is therefore evident that the neonatal calf model can prove useful as an *in vivo* platform for future exploration into pathways that specifically regulate innate immune responses during RSV infection.

Gamma delta T cells have been suggested to play a role in innate immunity to RSV based on their recognition of unprocessed and non-protein antigens independent of MHC restriction and their localization in epithelial tissues at the host-environmental interface [45]. In humans and mice, the frequency of γδ T cells in circulation or within secondary lymphoid tissues is low, generally representing less than 5-10% of the circulating peripheral lymphocyte population [46]. However, in ruminants, γδ T cells are significantly more abundant, representing up to 70% of the circulating peripheral blood lymphocytes in very young animals [47,48]. Given their low frequency and the difficulty in obtaining sufficient cell numbers, relatively few studies exist which have examined the role of γδ T cells during hRSV infection. A study in patients hospitalized for hRSV bronchiolitis revealed a significant reduction in the numbers of γδ T cells circulating in the blood compared to control patients. Interestingly, this reduction was more pronounced in patients with severe disease [49]. Aoyagi *et al.* recently demonstrated that γδ T cells isolated from the blood of hRSV-infected infants produce less IFNγ and more IL-4 in response to mitogen stimulation than equivalent cells isolated from rotavirus-infected infants [50], suggesting that γδ T cells may be susceptible to the Th2-skewing often associated with hRSV infection (see section on Adaptive immunity to RSV). Data from mice suggests that γδ T cells contribute to immunopathology at the site of infection, as depletion of γδ T cells prior to challenge with hRSV results in reduced lung inflammation and disease severity [51]. However, γδ T cells may play a role in limiting viral replication in the lungs as their depletion also resulted in an increase in peak viral titers [51].

Despite their abundance in circulation in the blood of ruminants, studies of γδ T cells in the bovine during bRSV infection are also relatively scarce. A study by Taylor *et al.* reported that depletion of WC1^+^ γδ T cells from neonatal gnotobiotic calves infected with bRSV had little effect on clinical signs or viral clearance after 10 days of infection, but resulted in a significant increase in IgM and IgA in the bronchiolar alveolar lavage [52]. Similarly, γδ T cell depletion does not yield changes in macroscopic or microscopic lesions in the lungs of bRSV-infected calves compared to non-depleted control animals [53]. The results of these studies are difficult to interpret however, as the experimental numbers are very small and the infection failed to yield any clinical signs. Recent results from our laboratory suggest that bovine γδ T cell subsets respond to *in vitro* and *in vivo* BRSV infection with pro-inflammatory chemokine and cytokine production [54], suggesting γδ T cells may have a role in recruiting effector cells to the site of infection. Together, there appears a potential role for γδ T cells in altering lung pathology and viral clearance following both hRSV and bRSV infection, but much remains to be examined including the potential innate role of γδ T cells in regulating early virus recognition and lymphocyte recruitment. 

## 7. Adaptive Immunity to RSV

The development of an adaptive immune response is required for the control and clearance of established RSV infections. Following infection, humans and cattle mount virus-specific antibody and T cell responses; however, these responses are weak and transient, as both species can be continuously re-infected throughout life. Clearance of RSV infection is primarily mediated by CD4 and CD8 αβ T cells. T cell responses are directed at epitopes within several RSV proteins including the N, M, NS2, M2-1, F and G [55]. The F and G proteins are the major HLA class II restricted targets in both humans and cattle [55,56], with the F protein of hRSV being the most thoroughly studied and described to contain multiple antigenic regions [57]. To our knowledge, there are currently no specific BoLA class II epitopes defined for bRSV in the bovine.

While important in anti-viral immunity, the RSV-specific CD4 T cell response is also thought to contribute to disease pathogenesis and damaging immunopathology [58,59,60]. CD4 T cells, and the cytokines they produce, are key in shaping the nature of the adaptive virus-specific immune response. HRSV infection induces a mixed Th1 and Th2-type cytokine response [61]. Production of IL-12 by dendritic cells, and early IFNγ is required for the priming of an effective Th1 type cytokine response; however, hRSV has been shown to interfere with dendritic cell cytokine production and their ability to initiate the development of a Th1 response [56,62,63]. The ensuing Th2 polarized response leads to increased disease severity and lung injury, and is thought to block the development of an effective CD8 T cell response during both primary and secondary challenge [56,64,65,66]. Much of our understanding about the adaptive immune response to hRSV is derived from small rodent models; however, evidence from humans, while limited, suggests a similar mechanism of disease pathogenesis. Infants infected with hRSV exhibit Th2 polarization with increased IL-4 production in the lungs and the establishment of eosinophilia [67,68]. Interestingly, eosinophilia was also apparent in the lungs of infants that exhibited vaccine enhanced disease after receiving a formalin-inactivated hRSV vaccine (see section on RSV Vaccines). However, cytokine balance and the resulting disease outcome likely depends upon genetic background, as some infants have also been shown to contain significant numbers IFNγ-producing cells in the lungs, and these increased levels did not correlate to disease severity or outcome [69].

Like humans, calves infected with bRSV develop a mixed cytokine response, but favor the development of a Th2-type immune response following infection. Studies of the cells and lymph fluid from bRSV infected calves reveal enhanced IL-4 and IL-13 production in the serum and tissues as early as day 4 post infection, and increased serum levels of virus-specific IgE, indicating the establishment of a Th2-type response [70,71,72]. Calves also develop IFNγ producing cells and levels of the cytokine increase in the serum, but, as with humans, neither cell numbers nor IFNγ levels correlate with positive disease outcome [73]. Evidence from humans has suggested that, due to the Th2 nature of the anti-viral immune response, hRSV infection may predispose children to the development of allergies and asthma later in life [74,75]. Interestingly, bRSV infection in calves has also been shown to predispose to allergic sensitization [76,77]. Gershwin *et al.* demonstrated that exposure to the model allergen ovalbumin during bRSV infection resulted in significantly increased levels of IL-4, IL-13 and ovalbumin-specific IgE compared to uninfected control calves [77]. 

Cytotoxic CD8 T cells play a critical role in the control and clearance of RSV infection. Infection of human infants results in a significant influx of activated CD8 T cells into the airways [78], and calves infected with bRSV exhibit increased CD8 T cell infiltration in the lungs, trachea and nasopharynx [79]. Depletion of CD8 T cells from mice [59] or bRSV infected calves [53] results in more severe disease and increased and sustained viral shedding compared to non-depleted control animals. In humans, the hRSV-specific HLA class I response is primarily targeted against the M2, F and N proteins [80,81,82]. Bovine CD8 T cells target the M2, F, N and G proteins of bRSV [83,84]. Interestingly, while G-specific CD8 T cells are readily detectable in cattle, they have not been demonstrated in humans [85]. It has been reported that the strong Th2 skewing that occurs during RSV infection acts to inhibit the development of an efficient CD8 T cell response and prevent the establishment of long-lived memory [56]. Anecdotally, this is evidenced by the recurring infections that occur commonly in both humans and calves [16,18]. In the mouse model, hRSV-specific CTLs appear impaired in both cytokine secretion and cytotoxicity [56,86,87], while calves infected with bRSV exhibit a similar phenotype, displaying limited bRSV-specific cytotoxicity during primary infections and impaired memory responses following challenge or vaccination [88,89]. 

Humoral immunity plays an important role in defending the host from RSV infection. While not fully effective, maternal antibodies may provide some level of protection from severe RSV infection in both humans [90,91,92] and calves [93]; however, their presence has also been described to suppress the development of antibody and T cell responses during acute infection [94,95]. Although humans initiate responses to several proteins of hRSV, only antibodies that are reactive to the major surface glycoproteins F and G appear to be important for protection [96,97]. The F protein is highly conserved and the majority of the F specific response is cross-reactive, making it more important for protection across hRSV strains [98]. The G protein is more divergent, thus few antibodies cross-react between virus strains [98,99]. Further, the G-specific response is particularly inefficient and it has been proposed that this may be due to the secreted form of the G protein acting as a decoy for the host’s humoral response [100,101].

Like humans, calves mount antibody responses to several bRSV antigens, but the primary targets for protective humoral responses are the F, G and NP proteins [84]. bRSV-specific IgM and IgA can be detected in the nasal secretions and serum of bRSV infected calves as early as 8 days post infection [95]. BRSV-specific IgG2, the antibody isotype associated with a Th1 response, is not detected in the serum until 1-3 months post infection. As evidence of the cytokine skewing that occurs during RSV infection of calves, virus-specific IgG1, the isotype associated with a Th2 phenotype, is detectable in the serum starting at 13 days post infection [95]. In a separate study, virus-specific IgE, another antibody associated with Th2 skewing and airway hyperresponsiveness, was detectable in the serum concurrent with the development of clinical signs [71,72].

In humans, both neutralizing IgG and IgA are thought to have a role in protection from RSV. IgA is important in local immunity, particularly in the upper airways and nasopharynx, while serum IgG plays a significant role in protection of the lower airways. Adults who have been repeatedly infected with hRSV develop sustained high levels of IgA in nasal secretions which has been shown to prevent virus replication in the upper airways, regardless of serum Ig levels [102]. Further underlining the importance of both antibody types, passive immunization studies in rodents have shown that administration of neutralizing IgG provides complete protection from hRSV replication in the lungs, but not in nasal secretions [103,104,105]. However, the IgA response is often transient and neutralizing IgM and serum IgG are likely more important for long-term protection [94]. To date, the only licensed therapy available for hRSV infection in humans is the passive transfer of the virus-specific monoclonal antiserum, Palivizumab, which recognizes a conserved epitope in the F protein of hRSV. As proof of the importance of neutralizing antibodies in the immune response to hRSV, the antiserum is effective as a prophylactic and known to reduce disease severity in hRSV-infected infants [106,107,108]. 

## 8. RSV Vaccines

In the 1960s, a formalin-inactivated hRSV vaccine was prepared and tested in infants and children. The Bernett strain of hRSV was initially propagated in human embryonic kidney cells and passaged in vervet monkey kidney cells. The infected cells were inactivated with formalin and concentrated by ultracentrifugation and alum precipitation. This preparation was known as lot 100. Lot 100 was administered as two or three intramuscular doses separated by 1 to 3 months to infants and children between 2 months and 7 years of age. Lot 100 not only failed to protect against hRSV disease, but also induced an exaggerated clinical response to hRSV infection in infants who were hRSV näive before vaccination [109,110,111]. Many vaccinates were hospitalized with LRI; in one study, the hospitalization rate of vaccinates approached 80% compared to 5% in controls [111]. Tragically, two infants who received lot 100 died following hRSV infection, one at 14 months of age and the second at 16 months of age [111]. HRSV was readily isolated from the lower respiratory tracts of these infants, whose lungs also contained eosinophilic infiltrates. The disastrous results of those clinical trials are still felt five decades later. To date there is no approved vaccine.

There are multiple vaccines currently marketed for bRSV (killed and modified-live) that are generally provided as part of multivalent products. As with humans, similar vaccine-enhanced disease has been reported from two cases of natural bRSV infections in calves [112,113]. In the former case, an outbreak of respiratory tract disease among 5- to 7-mo old calves on a beef-fattening farm in the Netherlands started two days after administration of a modified-live bRSV vaccine [112]. The disease was severe among vaccinates, but absent in non-vaccinated calves 8 mo of age or older. In the latter case, 30% of 8-mo old Belgian Blue calves vaccinated with a beta-propiolactone inactivated bRSV vaccine died during a naturally occurring bRSV outbreak. Interestingly, no deaths were recorded among younger calves not vaccinated [113]. Experimentally, vaccine-enhanced disease has been reproduced in some studies of calves vaccinated with formalin-inactivated bRSV preparations [114,115], but not in others [116,117]. Where bRSV vaccine-enhanced disease has been observed, there is a bias toward a Th2-like response characterized by increases in serum IgE [114,118] and eosinophils in lavage fluid [114].

Given the significant disease burden associated with hRSV and bRSV infections, there is a profound interest in developing new and more efficacious vaccines for both species. RSV vaccine development in both cattle and humans has been the topic of several recent and excellent reviews [55,101,119]. As such, we will only briefly describe some of the approaches currently being pursued in the field.

RSV and its target populations pose several obstacles with respect to vaccine development, particularly the need to vaccinate populations with immature immune systems, to induce a response in the face of maternal antibodies, and to induce an appropriate, robust and long-lasting immune response. To address these challenges, a variety of new hRSV vaccines have recently been tested in animal models and, in some cases, clinical trials. The primary focus of these new platforms is to induce a more robust response using actual replicating agents, as opposed to the use of inactivated virus, and to induce Th1-type CD4 T cell response through the use of known CD4 T cell targeted proteins or Th1 activating adjuvants. Amongst these new candidates are synthetic and subunit vaccines that target whole or fragments of the F, N, G and M proteins, singly or in combination. Other examples include the use of live attenuated hRSV, such as strains lacking nonessential genes such as the NS1, NS2, SH, G or M2-2 genes and/or cold-passaged temperature-sensitive mutants [55,119]. One such temperature-sensitive mutant, which lacks the SH gene and contains a mutation in the L gene, proved promising in early phase I trials but lacked efficacy during phase II clinical trials in seronegative infants [120,121]. The stability of the mutant has recently been improved and shown success in trials using seronegative chimpanzees [121]. The use of nanoparticles, virosomes and virus-like particles, as well as vector-based approaches using DNA, virus or bacterial-based vectors that express hRSV proteins have all proven promising [55,119]. One of the vectors that has shown potential is a chimeric bovine/human parainfluenza virus type 3-based vaccine that has been engineered to express the hRSV F protein [122]. This vaccine has proven safe in phase I clinical trials and is currently being tested in a large international clinical trial in infants [123,124]. 

Although there is widespread use of bRSV vaccines in calves, their efficacy is controversial and there is a definite need for improved technologies. Researchers are currently studying many of the same approaches as described for hRSV, including the use of subunit based vaccines and live attenuated bRSV [55]. One promising example reported by Valarcher *et al.* reported the success of two bRSV strains, one devoid of NS1 and the other lacking NS2 [125]. Calves vaccinated with either deletion mutant exhibited a robust virus-specific antibody and CD4 T cell response and were protected against virus challenge [125]. Interestingly, the NS2 mutant was more effective than its counterpart. Also being extensively pursued against bRSV is the use of new adjuvants coupled with inactivated bRSV or subunit vaccines. Amongst those showing promise is the use of CpG containing oligodeoxynucleotides (CpG-ODN) [126], and immunostimulating complexes (ISCOMS) [127,128]—both of which induce a robust Th1 skewing. ISCOMS are multimers composed of cholesterol, phospholipids, proteins and *Quillaja* saponins. Recent studies by Hagglund *et al.* have described the ability BRSV-ISCOMs to successfully induce bRSV-specific cellular and humoral responses and protect from virulent bRSV challenge in neonatal calves aged 3-8 weeks. Interestingly, this protection was robust despite the presence of significant levels of maternally derived antibodies [127,128].

## 9. Potential Role of Vitamin D as an Immunomodulator During RSV Infection

Recent evidence has suggested a role for vitamin D in hematopoietic cell differentiation and immune function [129,130]. It is known that the nuclear vitamin D receptor (VDR) and the enzymes responsible for activation (CYP27B1; 1α-hydroxylase) and degradation (CYP24A1; 24-hydroxylase) of vitamin D are expressed in subsets of immune cells. Moreover, several aspects of immune regulation are modified by the actions of vitamin D [129,131,132,133,134]. Based on these data and epidemiological evidence suggesting a connection between inadequate vitamin D levels and respiratory tract infections [135,136,137], there has been an examination of the ability of vitamin D to modulate the response to *in vitro* respiratory infections. In the case of hRSV, it was shown that treatment of cultured respiratory epithelial cells with 1,25(OH)_2_D_3_ decreases viral induction of pro-inflammatory gene expression [138]. In spite of the reduction of antiviral IFN-β, there was no concomitant increase in hRSV replication, suggesting that providing adequate vitamin D could reduce inflammation while maintaining antiviral activity.

We examined the influence of vitamin D status on the response to bRSV experimental challenge in calves [31]. Calves with high or low circulating 25(OH)D_3_ levels were challenged with RSV and subsequently, lung tissue samples examined at day 7 post infection. We showed, for the first time *in vivo*, that bRSV infection induced expression of the VDR and associated hydroxylase enzymes in the lung. Importantly, gene expression levels of pro-inflammatory cytokines were not suppressed in the presence of this induced vitamin D regulatory network, but rather specific pro-inflammatory cytokines were elevated in the high vitamin D group compared to the low vitamin D group of calves. Further examination of the potential effects of vitamin D status on bRSV disease resolution would require longer-term studies with immunologically sufficient and deficient vitamin D levels. Thus, preliminary bRSV challenge studies have been conducted with groups of calves with circulating levels considered sufficient (40–60 ng/ml) or deficient (<20 ng/ml) in 25(OH)D_3_. To date, we have not observed an alteration in the clinical course of bRSV as a result of providing differing levels of vitamin D supplementation.

## 10. Summary

In recent years, we have made significant progress in our knowledge of both bRSV and hRSV and the way each interacts with its respective host. To date, the disease process and lesions, as well as many aspects of the innate and adaptive immune response described during bRSV infection in the bovine parallel those described in human patients. See Table 1 for a summary of common features between bRSV and hRSV infection. From these data, we suggest that cattle serve as an excellent model for studying hRSV infection in humans. The field is currently pursuing several new approaches and vaccine platforms in both species that hold great promise for the future. Despite this, however, considerable challenges await with respect to our understanding of the RSV-specific immune response and the development of effective vaccine strategies. 

**Table 1:** Features common to hRSV in infants and bRSV in calves.

Feature	Human RSV	Bovine RSV	References
Age-dependency	More prevalent in children <2 yrs old	More prevalent in calves <6 mo old	[16,17]
Seasonal periodicity	More common in fall and winter	More common in fall and winter	[18]
Histopathology	Bronchiolitis; interstitial pneumonia;	Bronchiolitis; interstitial pneumonia;	[31,33,34,35]
	Prominent neutrophil and macrophage infiltration	Prominent neutrophil and macrophage infiltration	
CXC chemokines	CXCL8 (IL-8) upregulated	CXCL8 (IL-8) upregulated	[31,139]
Adaptive immunity	Th2 cytokine bias	Th2 cytokine bias	[67,68,70,71,72]
Vaccine-enhanced disease	Observed with formalin-inactivated vaccine	Observed with formalin-inactivated vaccine	[109,110,111,112,113]
